# In Silico Evaluation of Potential Hit Molecules Against Multiple Serotypes of Dengue Virus Envelope Glycoprotein

**DOI:** 10.3390/molecules30061268

**Published:** 2025-03-12

**Authors:** Aadhil Haq, Samavath Mallawarachchi, Aiden Anderson, Leily Khaleghi, Lasan Manujitha, Sandun Fernando

**Affiliations:** Department of Biological and Agricultural Engineering, Texas A&M University, College Station, TX 77840, USA; aadhil.haq@tamu.edu (A.H.); samavath@tamu.edu (S.M.);

**Keywords:** antiviral, dengue virus, computational drug discovery, serotypes, envelope protein inhibitor

## Abstract

Dengue Fever, a widespread mosquito-borne disease caused by the dengue virus (DENV), poses a major health threat in tropical and subtropical regions worldwide, resulting in millions of infections yearly. Severe cases of dengue fever have a mortality rate of around fifteen percent. Currently, there are no antiviral treatments for this disease and the only FDA-approved vaccine has been known to have adverse effects, especially in children. Thus, there is an urgent need for new therapeutics for Dengue fever. The largest issue with developing an antiviral treatment is that DENV has four serotypes that each differ slightly enough to pose problems with one compound inhibiting all four. This study addresses that challenge to some extent by focusing on in silico screening of potential hits targeting the envelope glycoprotein, which is relatively conserved across these four serotypes. Using pharmacophore screening and in silico evaluation of ligands, we identified compounds which could potentially have high affinity to the envelope glycoprotein for two of the four DENV serotypes. These in silico results were validated experimentally using bio-layer interferometry. These findings lay a foundation for in vitro analysis and hit-to-lead studies, advancing the development of antivirals that can inhibit multiple serotypes of the dengue virus.

## 1. Introduction

Dengue fever is one of the most severe and fastest-spreading mosquito-borne viral diseases worldwide. According to the World Health Organization (WHO), about half the world’s population is now at risk of dengue, and 100–400 million people are being infected every year, with 21,000 deaths worldwide [1,2]. Some researchers have estimated that the number of infections is even higher, due to under-reporting in African regions [3]. The incidence of dengue has increased exponentially over the last few decades, and currently, dengue is endemic in 112 countries in the world [2,3,4]. Clinical manifestations of dengue range from asymptomatic infections to severe and life-threatening conditions including dengue hemorrhagic fever and dengue shock syndrome. Severe dengue is characterized by increased vascular permeability, plasma leakage, and hemorrhagic phenomena, which can lead to hypovolemic shock and multiple organ failure [4,5,6].

Dengue virus (DENV) is a positive-sense, single-stranded RNA virus belonging to the genus *Flavivirus*, which also includes West Nile, Zika, and tick-borne encephalitis viruses. This viral genome encodes three structural and seven non-structural proteins and can be categorized into four serotypes (DENV1-4) based on the differences between these proteins. DENV is transmitted through the bite of infected mosquito vectors belonging to *Aedes aegypti* and *Aedes albopictus* species [6].

Currently, there are no clinically approved drugs for the treatment of dengue, and the treatment is generally limited to supportive care, including intravenous fluid supplementation and providing pain relievers [7,8,9,10]. While some antivirals, such as celgosivir and doxycycline, have achieved dengue viral inhibition in in vitro tests, those compounds have shown only limited efficacy in clinical trials [11,12,13]. Dengvaxia is a vaccine approved by the FDA for dengue prevention, while several other vaccines, such as CYD tetravalent vaccine, have been used in other countries [14,15,16,17]. However, there are concerns about the vaccines increasing the risk of severe dengue in some individuals, and some studies have shown that Dengvaxia has lower efficacy against DENV1 and DENV2 serotypes [18,19,20]. Thus, there is a critical need to develop new antivirals targeting DENV.

One of the main challenges in developing antivirals targeting DENV is developing drugs effective against all four serotypes. The four serotypes of DENV differ slightly enough to pose problems with one compound inhibiting all four. While many groups have attempted to develop antiviral drugs effective against one or two serotypes, only a handful of groups have been successful in showing inhibitory results across all four serotypes, and almost all these molecules are in the early stage of research and development [7,18,21,22,23,24,25]. The presence of multiple serotypes is also a significant challenge in vaccine development, since vaccines that boost immunity against only some of the serotypes can increase the risk of severe dengue in a subsequent infection by a different serotype [14,19]. Due to their conservation across serotypes, the envelope protein (E) and the non-structural proteins NS3 and NS5 have been major targets of interest in drug development targeting DENV [22,26,27].

This study presents an in silico evaluation of antivirals targeting the E protein of multiple DENV serotypes. The E protein is the most conserved among structural proteins and is vital in viral entry and membrane fusion during infection. Furthermore, the E protein is the most accessible protein of DENV for any drug molecule. Therefore, inhibiting cell attachment and membrane fusion via targeting the E protein is a feasible strategy to neutralize the threat posed by DENV [28]. The E protein consists of three distinct domains and takes a dimeric conformation in the mature virus. The Envelope Protein Domain III (ED-III) consists of the amino acids vital for receptor binding during viral entry. A few amino acids within the fusion loop are required for viral maturation and membrane fusion, while a pocket known as the β-OG within the ED-I and ED-II boundary is highly conserved across all four serotypes, and has been widely accepted as an appropriate target for drug discovery [20,29,30,31]. In this study, we present the use of in silico tools to screen antivirals targeting this conserved region in the envelope protein.

## 2. Results and Discussion

### 2.1. Docking of the Controls and Pharmacophore Screening

**Figure 1:**
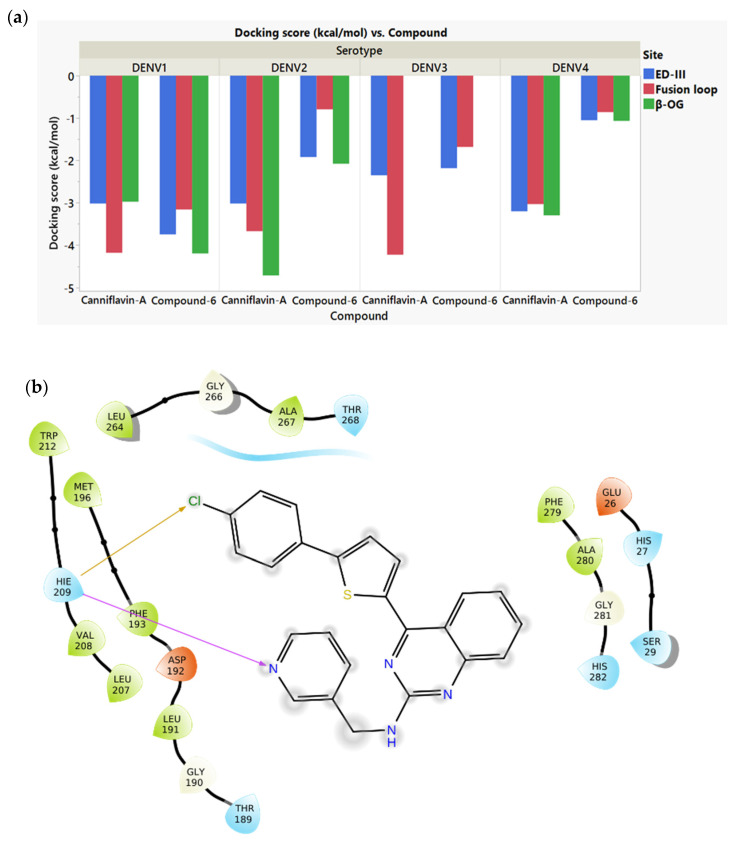

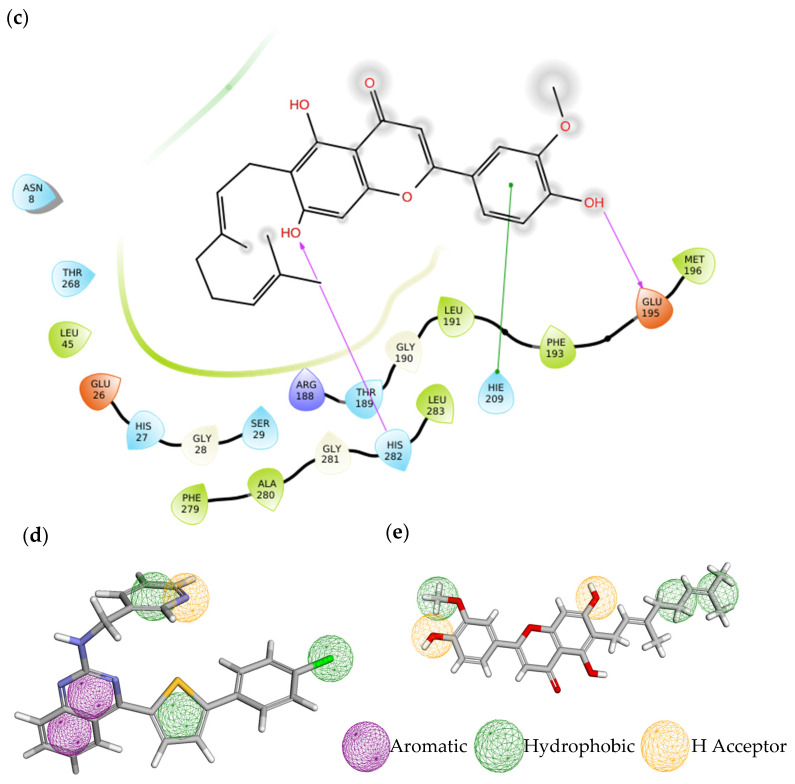
(**a**) Docking scores of Canniflavin A and Compound **6**, showing the affinities to the 3 binding sites of the E protein: fusion loop, EDIII, and β-OG. (**b**) Ligand interaction diagram of Compound **6** docked to the β-OG binding site of DENV1 Envelope protein. (**c**) Ligand interaction diagram of Canniflavin A docked to the β-OG binding site of DENV1 Envelope protein. (**d**) Pharmacophore features chosen for compound screening of Compound **6** analogs. (**e**) Pharmacophore features chosen for compound screening of Canniflavin A analogs. Pink arrows show hydrogen bonds, the yellow arrows show halogen bonds, and the green lines show pi–pi stacking interactions.

Based on the literature, we selected two control molecules that have been reported to inhibit DENV E protein: Compound **6**, a synthesized molecule that has demonstrated an IC50 value of 119 nM against DENV2 E protein [26], and Canniflavin A, a naturally occurring molecule derived from the *Cannabis sativa* plant [27]. To assess the specificity of literature-derived control molecules, we initially performed docking simulations at three distinct binding sites: β-OG, ED-III, and fusion-loop. The docking results for the control molecules on these sites of all DENV serotypes are given in Figure 1a. As depicted in Figure 1a, the docking scores for at least one of these control molecules were consistently lower (more negative indicating stronger affinity) within the β-OG pocket for all serotypes, except DENV3. DENV3 showed a different behavior with Canniflavin-A showing specificity towards the Fusion Loop and Compound **6** showing specificity towards ED-III. Since β-OG is reported to be the most conserved domain [20,29,30,31], and the control molecules generally showed stronger binding to β-OG in three of the serotypes compared to the other two sites, β-OG was selected as the binding site for subsequent steps of the virtual screening, meaning that the pharmacophore features considered for virtual screening were based on the control molecules binding to the β-OG pocket.

To further elucidate the molecular interactions and identify key pharmacophore features, ligand interaction diagrams were generated for the control molecules (Figure 1b,c). Virtual screening was performed using six pharmacophore features for each control molecule. For Compound **6**, chlorobenzene and benzene groups showed hydrophobic interactions, while the N in the pyridine group formed a hydrogen bond. Therefore, the hydrophobic feature was toggled on in the Pharmitt platform at the appropriate locations for thiophene, phenylenediamine, and benzene regions within the structures that were exposed to more hydrophobic residues, as shown in Figure 1b,c [32]. The hydrogen acceptor feature was toggled on for the pyridine N. Similarly, with Canniflavin A, the hydrogen donor feature was toggled on for the hydroxyl groups in the methoxy phenol and the dihydric phenol groups, as per the ligand interaction diagram Figure 1c. The pharmacophore features based on Compounds **6** and Canniflavin A that were included in the Pharmitt platform are given in Figure 1d,e, respectively.

**Figure 2:**
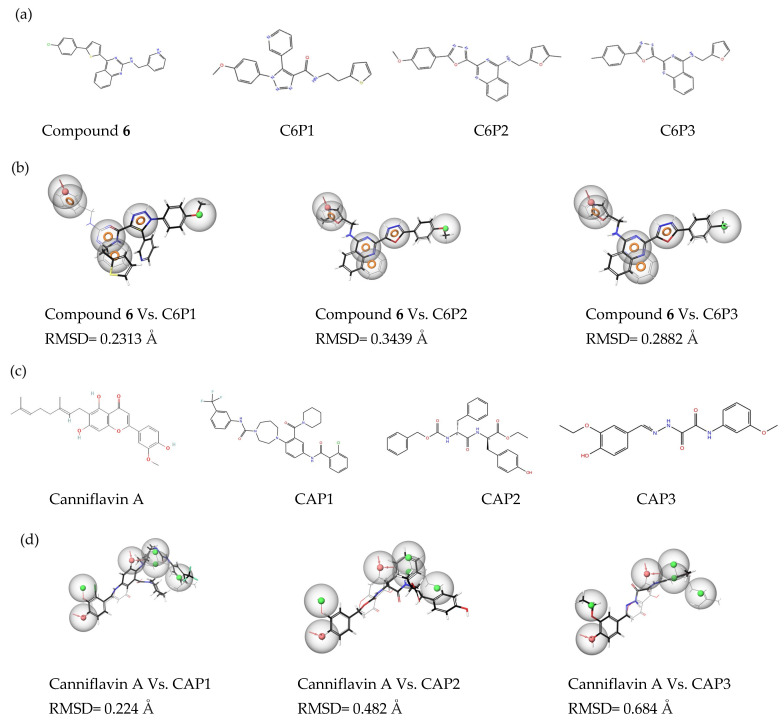
(**a**) Chemical structures of Compound **6** and its analogs. (**b**) Aligned pharmacophore features of Compound **6** analogs and their RMSD with reference to Compound **6** pharmacophores. (**c**) Chemical structures of Canniflavin A and its analogs. (**d**) Aligned pharmacophore features of Canniflavin A analogs and their RMSD with reference to Canniflavin A pharmacophores.

The top three compounds from each screening based on the lowest RMSD were selected for docking studies [33]. The compounds screened using Compound **6**’s pharmacophore features were dubbed C6P1, C6P2, and C6P3, and the compounds screened using Canniflavin A’s pharmacophore features were dubbed CAP1, CAP2, and CAP3. The selected compounds’ structures and the aligned pharmacophores along with the RMSD values are shown in Figure 2. 

### 2.2. Docking of the Screened Compounds

The selected compounds from the pharmacophore screening were docked on the E protein. Two additional compounds from the literature, Paclitaxel, and Malacitanolide, suggested as promising inhibitors against DENV, were included in the docking study [34]. The PubChem IDs of the molecules are included in the Supplementary Table S1. The chemical structures of Paclitaxel and Malacitanolide are shown in Figure S1.

The docking scores of the screened compounds on the selected binding sites of the E protein of all serotypes of DENV are given in Table 1. Among the Compound **6** analogs, C6P3 has shown the highest specificity, showing the strongest binding at the β-OG in all serotypes except DENV3. However, C6P3 has shown a stronger affinity compared to Compound **6** at DENV3 β-OG. In comparison, C6P1 and C6P2 showed lower specificity across serotypes.

All Canniflavin A analogs have shown similar binding patterns, preferring the Fusion loop in DENV1 and DENV4, ED-III in DENV3, and β-OG in DENV2. Among those three molecules, CAP1 showed the most negative docking scores at the preferred binding site for all serotypes except DENV1. CAP1 also showed the strongest affinity among the analogs towards the β-OG of DENV2, which is considered the most clinically significant serotype due to the high prevalence and mortality rate [35,36]. In general, CAP1 and C6P3 showed a stronger binding affinity than the controls at β-OG of most serotypes and were selected for the molecular dynamics studies.

### 2.3. ADMET Properties

The predicted ADMET properties of the selected compounds are summarized in Table 2. All compounds except Canniflavin A and Paclitaxel showed a high blood–brain barrier (BBB) penetration probability. In vivo and in vitro studies have shown DENV to have the capability to affect cells from the BBB [37]. However, since the peripheral activity of DENV is higher, not having BBB activity can still be suitable for an antiviral drug candidate against DENV [38]. All molecules except Canniflavin A show positive human intestinal absorption, which suggests a favorable oral bioavailability profile. Low solubility and absorption are commonly observed in flavonoids, and the literature indicates the availability of strategies for enhancing oral bioavailability [39,40]. Considering aqueous solubility, Canniflavin A, Compound **6**, and CAP1 molecules indicate values below the ideal range of −1 and −4, suggesting challenges in formulation and absorption, often needing formulation enhancement strategies. Only C6P1 has demonstrated positive Caco-2 permeability, a predictive measure of passive absorption through the intestinal wall. This suggests that most compounds may rely on alternative transport mechanisms or are less likely to passively permeate through intestinal cells, potentially influencing their formulation and dosage design. C6P2 displayed AMES mutagenicity, while all other compounds displayed a high probability of not having AMES mutagenicity and were considered non-carcinogenic in the prediction. The acute oral toxicity values displayed moderate to low values comparable with approved drug compounds such as Paclitaxel and inhibitors such as Malacitanolide.

The results of the drug likeliness of all molecules based on Lipinski’s rule of 5 are presented in Table 3. All molecules except Paclitaxel and CAP1 had no more than one violation. CAP1 had two violations, suggesting it may have low bioavailability, which agrees with ADMET results [41].

### 2.4. Molecular Dynamics Simulation of the Selected Molecules

**Figure 3:**
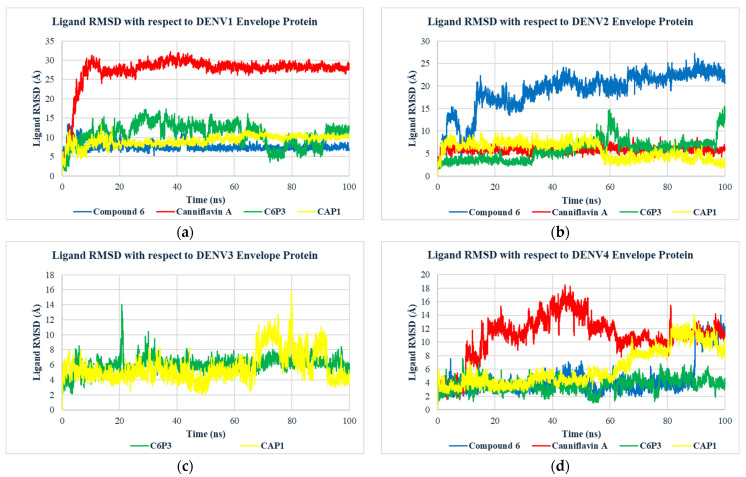
Variation in Ligand RMSD with time for the selected compounds bound to the β-OG site of (**a**) DENV 1, (**b**) DENV2, (**c**) DENV3, and (**d**) DENV4 envelope protein.

The binding of selected compounds and controls on the β-OG pockets of the four serotypes was further analyzed using molecular dynamics (MD) simulations. The stability of the MD simulations was determined using RMSD analysis (Figure 3). Compound **6** showed the lowest RMSD and most stable binding for DENV1, but did not show stable binding to any other serotype. Canniflavin A only showed stable binding to DENV2, demonstrating large fluctuations in DENV1 and DENV4. In comparison, CAP1 and C6P3 showed more stable binding across multiple serotypes. CAP1 showed relatively stable binding to all serotypes except DENV4. CAP1 showed the most stable binding for DENV2, achieving stability after 60 ns at an RMSD less than 4 Å, which is indicative of stable binding [42,43]. C6P3 showed the lowest RMSD for both DENV3 and DENV4 and demonstrated relatively stable binding to DENV2 compared to Compound **6**. Therefore, the RMSD analysis suggests that CAP1 and C6P3 can maintain stable binding with multiple dengue serotypes.

Long-term binding strength during the simulation was evaluated by mean MM-GBSA for the last 50 ns of the trajectory. The results are summarized in Table 4. Compound **6** showed strong binding to DENV1 but significantly weaker binding to other serotypes, which agrees with the RMSD analysis. Similarly, Canniflavin A showed strong binding only to DENV2. CAP1 showed the best binding across all four serotypes. It showed a binding energy of −72.26 kcal/mol to DENV1, which indicates a comparatively strong binding [43]. It also demonstrated moderately strong binding to DENV2 and DENV4. C6P3 also showed moderately strong binding to all serotypes except for DENV2. Typically, binding energies between −30 kcal/mol and −50 kcal/mol are considered moderate and binding energies more negative than −50 kcal/mol are often considered strong [44,45,46].

**Figure 4:**
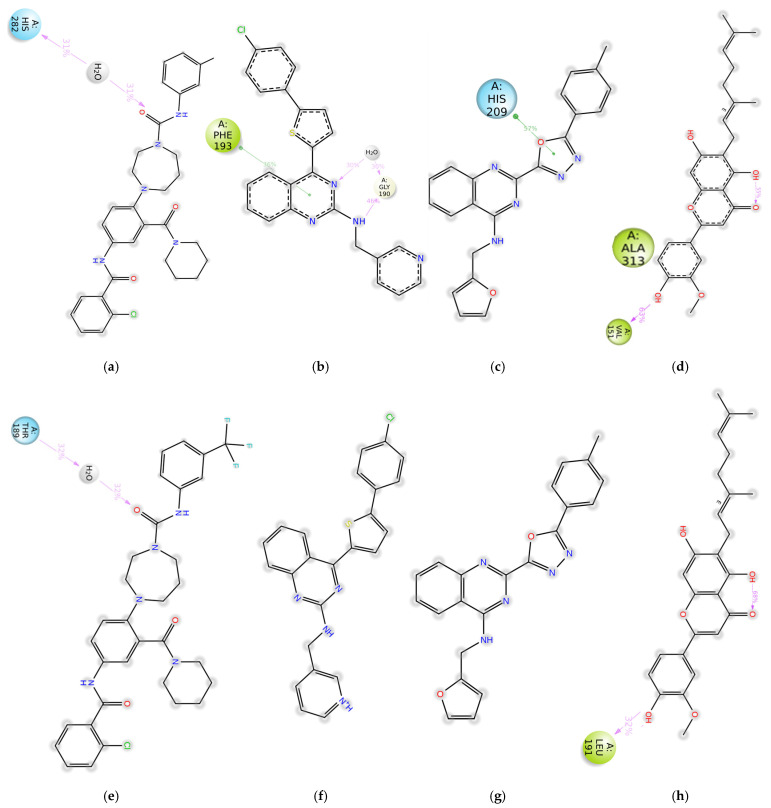
Simulation interaction diagrams of DENV 1 with (**a**) CAP1, (**b**) Compound **6**, (**c**) C6P3, (**d**) Canniflavin A, and DENV2 with (**e**) CAP1, (**f**) Compound **6**, (**g**) C6P3, and (**h**) Canniflavin A. Pink arrows show hydrogen bonds, and the green lines show pi–pi stacking interactions. Intramolecular interactions have been denoted usin dotted line arrows. The time fraction where the interaction was maintained during the MD simulation is mentioned on the arrows/lines.

As shown in Figure 4, the fraction of simulation time during which any residue in DENV2 interacted with Compound **6** or C6P3 did not exceed 30%. In contrast, Cannflavin A and CAP1 maintained stable hydrogen bond interactions with at least one residue of the DENV2 E protein. In the case of DENV1, at least one receptor residue exhibited an interaction fraction of 30% or higher with all four compounds throughout the simulation. CAP1, Compound **6**, and Canniflavin A maintained hydrogen bond interactions, while Compound **6** and C6P3 displayed pi–pi stacking interactions. Complete interaction data from the simulations are available in the Supplementary Information section and can be accessed at https://zenodo.org/records/14004458 (accessed on 12 February 2024). Based on the simulations, CAP1 was selected for experimental validation using BLI.

### 2.5. Binding Kinetics of CAP1 and Paclitaxel

The binding kinetics of CAP1 and Paclitaxel onto immobilized DENV1 and DENV2 E proteins in real time were obtained using BLI. These two serotypes were selected for experimental validation, since DENV2 is the clinically most important serotype, and DENV1 was used for pharmacophore screening. The affinity constant (KD), association rate constant (k_a_), and dissociation rate constant (k_d_) are summarized in Table 5. The results obtained for DENV2 as the receptor and CAP1 as the analyte did not generate results with dose dependence, while one of the trials with Paclitaxel as the analyte also did not show dose dependence (Figures S6, S8 and S9). Therefore, one- or two-point measurements were reported for those trials. The results indicate that the CAP1 molecule showed micromolar-level affinity to both DENV1 and DENV2, while there was nanomolar-level affinity in the case of Paclitaxel. It is interesting to note that the affinity constant and dissociation rate followed a similar pattern to results seen in the MM-GBSA scores of the MD trajectory for CAP1 analyte.

## 3. Materials and Methods

### 3.1. Protein and Ligand Structures

The envelope protein experimentally resolved structures of the 4 serotypes of the virus were obtained from the RCSB Protein Data Bank (PDB) (www.rcsb.org, accessed on 12 February 2024) and were used to carry out the preliminary simulation work: DENV1 (PDB ID: 7A3R) [47], DENV2 (PDB ID: 6WY1) [48], DENV3 (PDB ID: 1UZG) [49], and DENV4 (PDB ID: 7A3Q) [47]. The Protein Preparation Wizard in Schrödinger suite 2022-1 was used to prepare the protein structures in this study. During the process, all crystallographic water molecules were removed, and missing hydrogen atoms were added. The OPLS4 force field was used to optimize the geometry, and the protein was minimized to an energy minimum.

A common feature pharmacophore screening method (described further in the pharmacophore screening method section) was used to virtually screen potential drug molecules from the MolPort database of drug-like small molecules [50]. The ligands were prepared using the LigPrep module of Schrödinger suite 2022-1, and the OPLS4 force field was used for generating the ionization and tautomeric states at pH 7.0 ± 2.0 based on the Epik module of LigPrep.

### 3.2. Molecular Docking

**Figure 5:**
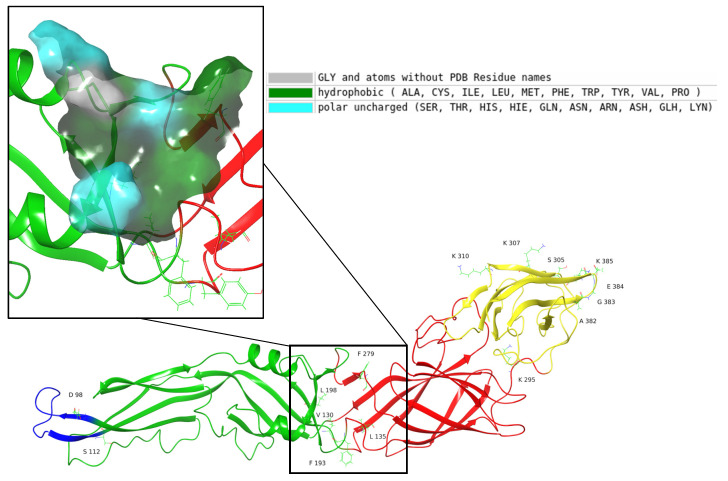
The 7A3R structure with important residues labeled. EDI is marked in red, EDII in green with the fusion loop region in blue, and EDIII in yellow. The square indicates the hydrophobic β-OG binding pocket, which is magnified to highlight the features of the binding pocket.

Ligands were docked on the receptors using Schrodinger Glide, where the docking process includes sampling the ligand’s position, conformation, and orientation, followed by assessing the energy of the ligand–protein interactions [51]. The receptor grid generation panel was used to produce the receptor grid for all the protein structures. Three receptor grid boxes per protein structure were created around the β-OG pocket, the ED-III, and the Fusion Loop. ED III—receptor binding pocket—was selected around K295, S305, K307, K310, A382, G383, E384, and K385, where the Serine (S) and Lysine (K) residues play a major role in attachment to the cell receptors. The hydrophobic β-OG pocket is surrounded by residues V130, L135, F193, L198, and F279. These hydrophobic amino acids are expected to show good binding with hydrophobic compounds. The E protein fusion loop is marked by the residue numbers D98–G112 [27,39,42,43]. The important residues selected are illustrated in Figure 5. The centroids of the above residues were used to create grid boxes of 20 Å size.

Glide ligand docking was carried out using the generated receptor grids. The standard precision option (SP) was used with flexible docking and default settings. The binding affinity between the ligand and receptor was evaluated based on the glide energy and docking score.

### 3.3. Pharmacophore Screening

Pharmit (http://pharmit.csb.pitt.edu, accessed on 15 February 2024), an online tool available to carry out the virtual screening of databases, was used to identify potential ligands using pharmacophore features [33]. Compound **6** and Canniflavin A were used as positive controls after confirming that they docked into the β-OG pocket of the envelope protein structures of the four serotypes, given the known interactions with this binding site based on literature evidence [26,27]. The receptor–ligand interactions identified key pharmacophore features, which were subsequently utilized to screen potential molecules in Pharmit’s MolPort database, containing over 64 million molecules. Two independent screenings were conducted, leveraging the receptor–ligand interactions of Compound **6** and Canniflavin A, to identify the top three compounds from each with the lowest RMSD deviations from the original pharmacophores. The DENV1 structure was chosen as the receptor due to the high conservation of the β-OG pocket across all four dengue virus serotypes. The relevant pharmacophore features were toggled as shown in Figure 1d,e in the Pharmitt platform to emphasize the key binding interactions displayed by the control molecule, refining the screening results. The selection of pharmacophore features is discussed in detail in the results section.

After this adjustment, the compounds were sorted based on the lowest RMSD (Root Mean Square Deviation) relative to the original molecules, allowing for the identification of new compounds with structural and binding characteristics similar to the known compounds [50].

### 3.4. Molecular Dynamics (MD)

The molecules that had the highest docking scores and glide energies were chosen in addition to the control molecules for the molecular dynamics simulations. As a secondary criterion, preference was given to molecules that showed a higher affinity for the β-OG pocket compared to the other two binding pockets (ED-III and Fusion Loop) across at least two serotypes. This approach aimed to increase the likelihood of identifying drug-like molecules with greater specificity.

Molecular dynamics (MD) simulations were performed using Desmond through the Schrödinger Maestro interface using Schrödinger suite 2022-1. These simulations were conducted on the docked protein–ligand complexes of the selected ligands docked to the β-OG pocket. The system was modeled within an orthorhombic simulation box filled with water molecules using the TIP3P solvation model, and Cl^−^ or Na^+^ ions were added to neutralize the overall charge of the complex. The default force field (OPLS4) was used to run the simulation of 100 ns using the NPT ensemble at 1.01325 bar and 300 K, with a recording interval of 20 ps. The Nose–Hoover chain method with a 1.0 ps interval served as the thermostat, and the Martyna–Tobias–Klein method with a 2.0 ps interval was used as the barostat. The molecular dynamics simulation results were analyzed using the Maestro simulation interaction diagram.

### 3.5. Prime MM-GBSA Binding Energy Calculations

A widely used computational approach for estimating the relative binding affinities of protein–ligand complexes is molecular mechanics combined with the generalized Born surface area (MM-GBSA) method [46]. This method calculates the free energy change during protein–ligand interactions. A greater change in free energy indicates a stronger interaction and higher complex stability. The more negative the free energy value, the greater the energy released during complex formation, signifying a stronger binding affinity. Using the MM-GBSA tools available in the Prime module of Schrödinger 2022-1, protein–ligand binding energies were calculated based on frames extracted from the molecular dynamics (MD) trajectory. Specifically, every 10th frame of the last 50 ns of the MD trajectory was analyzed to compute the relative binding free energy, ΔG_bind_, which was determined using the equation

ΔG_bind_ = E_complex(minimized)_ − (E_ligand(unbound,minimized)_ + E_receptor(unbound,minimized)_)

Here, ΔG_bind_ represents the computed relative binding free energy, accounting for the strain energy of both the ligand and the receptor.

### 3.6. Assessment of Absorption, Distribution, Metabolism, Excretion, and Toxicity (ADMET) Properties

AdmetSAR 2.0 is a tool available online used to analyze the ADMET (adsorption, distribution, metabolism, excretion and toxicity) properties of potential ligands (https://lmmd.ecust.edu.cn/admetsar2/, accessed on 20 Septermber 2024) [52,53]. Similarly, ProTox 3.0 is a web server for predicting the toxicity of chemicals (https://tox.charite.de/protox3/, accessed on 5 March 2025) [54]. The blood–brain barrier (BBB), human intestinal absorption (HIA), Caco-2 permeability, AMES mutagenicity, carcinogenesis, biodegradation, aqueous solubility, acute toxicity, LD50 value, hepatotoxicity, and fish aquatic toxicity results were predicted using these tools for all ligands. Lipinski’s rule of five was used to further evaluate the molecules based on the molecular weight being < 500 Da, the AlogP < 5, H-bond acceptors being < 10, and H-bond donors being < 5.

### 3.7. Viral E Proteins and Chemicals

The His-tagged E proteins of DENV1 and DENV2 were purchased from the catalog of Sino Biological, Houston, TX, USA. Two compounds were used as analytes in the bio-layer interferometry (BLI) assays. The CAP1 molecule was purchased from MolPort SIA, supplied by ChemDiv, Inc. (San Diego, CA, USA), and Paclitaxel was purchased from VWR International, LLC (Radnor, PA, USA), supplied by MP Biomedicals, LLC (Santa Ana, CA, USA).

### 3.8. Bio-Layer Interferometry (BLI)

The affinity measurements and binding kinetics of the compounds were assessed using BLI. The experiments were conducted on an Octet R4 (Sartorius, Bohemia, NY, USA) instrument with a 96-well plate. The Octet Anti-Penta-HIS (HIS1K) biosensor was used because the E proteins, purchased from Sino Biological, Houston, TX, USA, were His-tagged.

Initially, the biosensors were dipped in an assay buffer consisting of PBS (pH 7.4) and 0.5% DMSO for 60 s to establish an initial baseline. Two assays were performed to evaluate the two analyte molecules, Paclitaxel (positive control) and CAP1. A reference sample (assay buffer only) was included for baseline correction to account for non-specific binding [55]. Each experiment was performed independently in duplicate. Paclitaxel was chosen as the positive control for the BLI experiments due to the unavailability of a known direct antiviral against the DENV E protein, while literature-based evidence from a previous in silico study suggests that Paclitaxel is a known binder to the DENV2 E protein [34,56]. Data were analyzed using a double-referencing method and fitted to a 1:1 global fitting model. The kinetic constants were calculated using the Octet Discovery Analysis Studio 13.0 software.

Assay 1 was optimized for the analyte Paclitaxel. During the loading step, His-tagged E proteins were immobilized onto the biosensors for 240 s at a concentration of 10 µg/mL in PBS (pH 7.4) and 0.5% DMSO. A second baseline step of 30 s was performed by dipping the biosensors in a 1× kinetics buffer solution prepared from Octet Kinetics Buffer 10× (Sartorius, Bohemia, NY, USA). For the association (150 s) and dissociation (300 s) phases, the biosensors were dipped into the analyte solution and 1× kinetics buffer solution, respectively. Paclitaxel was tested at concentrations of 25 µM, 12.5 µM, and 6.25 µM, with the first well serving as a negative control (1× kinetics buffer without the analyte).

Assay 2 was optimized for the analyte CAP1. The loading step was 140 s, followed by association and dissociation phases of 100 s and 220 s, respectively. CAP1 was tested at concentrations of 50 µM, 25 µM, and 12.5 µM, with the first well serving as a negative control (1× kinetics buffer without the analyte). The biosensors were pre-hydrated in the assay buffer for 10 min at room temperature before the beginning of the assays [57].

## 4. Conclusions

In this study, an in silico approach based on pharmacophore screening, molecular docking, and molecular dynamics simulations was used to screen compounds with a high affinity towards the envelope (E) protein of all DENV serotypes. The in silico analysis of Canniflavin A, Compound **6**, and their analogs revealed interactions with the E protein of DENV across all four serotypes. Among the screened compounds, CAP1 and C6P3 demonstrated stronger and more stable binding to the β-OG pocket of the E protein across serotypes, compared to the control molecules Compound **6** and Canniflavin A. Molecular dynamics simulations and BLI experiments validated that CAP1 may serve as a viable candidate for targeting DENV’s E protein, a relatively conserved structural component critical to viral entry, with the possibility of broad-spectrum antiviral activity across serotypes. Further validation, including in vitro assays and pharmacokinetic analysis, is required before considering these molecules for clinical applications. Overall, this study provides valuable insights into potential antiviral compounds, which can contribute to the development of antivirals targeting all four DENV serotypes.

## Figures and Tables

**Table 1:** Docking scores for the strongest binding conformations of the screened molecules on the E protein of DENV serotypes and the structures of the molecules.

	DENV1			DENV2			DENV3			DENV4		
	β-OG	ED-III	Fusion Loop	β-OG	ED-III	Fusion Loop	β-OG	ED-III	Fusion Loop	β-OG	ED-III	Fusion Loop
Paclitaxel	-	−4.04	-	-	-	-	-	-	3.73	-	-	−2.41
Malacitanolide	−3.06	−3.38	−3.17	−3.28	-	−4.18	−1.70	−3.71	−3.03	-	−2.97	−3.32
Canniflavin A	−2.97	−3.02	−4.18	−4.72	−3.02	−3.67	-	−2.35	−4.23	−3.30	−3.20	−3.03
CAP1	−2.85	−2.66	−3.03	−4.44	−4.44	−2.48	−2.86	−4.64	−3.52	−3.26	−3.62	−4.16
CAP2	−3.50	−3.09	−3.77	−4.15	−3.81	−3.23	−3.02	−3.76	−3.09	−2.00	−3.22	−4.13
CAP3	−3.41	−3.39	−4.07	−3.41	−2.77	−2.97	−1.59	−4.56	−2.79	−3.01	−2.66	−4.01
Compound **6**	−4.20	−3.75	−3.16	−2.08	−1.92	−0.79	-	−2.18	−1.68	−1.07	−1.05	−0.86
C6P1	-	−2.76	−3.34	−4.12	−2.13	−2.52	-	−3.07	−3.16	−3.05	−2.77	−3.32
C6P2	−2.97	−3.64	−3.55	−4.00	−3.05	−3.89	−3.94	−3.31	−3.36	-	−2.18	−3.15
C6P3	−4.08	−3.57	−3.75	−4.03	−2.87	−2.73	−2.78	−4.52	−2.66	−4.05	−2.21	−2.74

**Table 2:** ADMET properties of the screened compounds predicted by AdmetSAR 2.0 and ProTox 3.0 server.

Name	BBB	HIA	Caco2 Permeability	AMES Mutagenicity	Carcinogenesis	Hepatotoxicity	Biodegradation	Aqueous Solubility LogS	LD50 (mg/kg)
Paclitaxel	-	+	-	-	-	-	-	−3.8727	134
Malacitanolide	+	+	-	-	-	-	-	−3.8155	1330
Cannflavin A	-	-	-	-	-	-	-	−4.4887	3919
CAP 1	+	+	-	-	-	-	-	−4.9531	678
CAP 2	-	+	-	-	-	-	-	−3.732	3120
CAP 3	-	+	-	-	-	-	-	−3.2751	5000
Compound **6**	+	+	-	-	-	-	-	−4.2568	1330
C6P 1	+	+	+	-	-	-	-	−2.8656	300
C6P 2	+	+	-	+	-	-	-	−3.2004	1000
C6P3	+	+	-	-	-	-	-	−3.1335	550

Abbreviations—BBB: blood–brain barrier; HIA: human intestinal absorption; Caco2: human colon adenocarcinoma cell. + symbol indicates a favorable result whereas the - indicates a deviation.

**Table 3:** Lipinski’s Rule of Five results.

Name	Molecular Weight (Da)	AlogP	Num. H-Bond Acceptors	Num. H-Bond Donors	Rule Violations
Paclitaxel	972.01	3.25	18	5	2
Malacitanolide	394.42	−0.1	8	3	0
Canniflavin A	436.5	5.82	6	3	1
CAP1	628.1	6.98	4	2	2
CAP2	490.56	3.52	6	3	0
CAP3	357.37	1.89	6	3	0
Compound **6**	428.95	6.69	5	1	1
C6P1	368.4	4.29	6	2	0
C6P2	413.44	4.87	8	1	0
C6P3	383.41	4.86	7	1	0

**Table 4:** Trajectory MM-GBSA binding energies for the selected compounds on the envelope proteins of the DENV serotypes.

Serotype	MM-GBSA Binding Energy (kcal/mol)			
	Compound 6	C6P3	Canniflavin A	CAP1
DENV1	−61.88434	−42.15755	−38.46022	−72.25684
DENV2	−33.57146	−33.80145	−57.20999	−47.91876
DENV3	0.00000	−51.60349	0.00000	−32.30574
DENV4	−47.78996	−59.98014	−36.47438	−53.62067

**Table 5:** Affinity and binding kinetics measurements of CAP1 and Paclitaxel to His-tagged DENV1 and DENV2 E protein.

Serotype	Molecule	KD (mM)	k_a_ (1/Ms)	k_d_ (1/s)
DENV1	Paclitaxel	(6.408 ± 1.272) × 10^−7^	(792.90 ± 157.90)	4.883 × 10^−7^
	CAP1	(7.371 ± 0.697) × 10^−4^	(3754.50 ± 1253.50)	(2.680 ± 0.663) × 10^−3^
DENV2	Paclitaxel	(6.635 ± 4.016) × 10^−7^	(1161.65 ± 703.35)	4.883 × 10^−7^
	CAP1	(1.371 ± 0.958) × 10	(355.79 ± 304.31)	(1.964 ± 0.765) × 10^−1^

## Data Availability

The original contributions presented in this study are included in the article/Supplementary Materials. Further inquiries can be directed to the corresponding author.

## Supplementary Materials

The following supporting information can be downloaded at: https://www.mdpi.com/article/10.3390/molecules30061268/s1. Table S1. PubChem IDs of selected ligands. Figure S1. Chemical structures of (a) Paclitaxel and (b) Malacitanolide. Table S2. ADMET profile of all the molecules used for the study. Table S3. BLI results summary. Figure S2. DENV1—Paclitaxel trial 1 BLI assay. Figure S3. DENV1—Paclitaxel trial 2 BLI assay. Figure S4. DENV1—CAP1 trial 1 BLI assay. Figure S5. DENV1—CAP1 trial 2 BLI assay. Figure S6. DENV2—Paclitaxel trial 1 BLI assay. Figure S7. DENV2—Paclitaxel trial 2 BLI assay. Figure S8. DENV2—CAP1 trial 1 BLI assay. Figure S9. DENV2—CAP1 trial 2 BLI assay.
