# Supplementary material for: In Silico Evaluation of Potential Hit Molecules Against Multiple Serotypes of Dengue Virus Envelope Glycoprotein
Source: Molecules. 2025 Mar 12;30(6):1268. doi: 10.3390/molecules30061268 (PMC11944462; doi:10.3390/molecules30061268)
Supplement: Supplementary file 1 [file molecules-30-01268-s001.zip › molecules-3492624-supplementary.pdf]

## Supplementary Information

## Supplementary Tables

*Table S1. PubChem IDs of selected ligands.*

| Ligand name | Pubchem ID |
|---|---|
| Paclitaxel | https://pubchem.ncbi.nlm.nih.gov/compound/36314 |
| Malacitanolide | https://pubchem.ncbi.nlm.nih.gov/compound/10739390 |
| Cannflavin A | https://pubchem.ncbi.nlm.nih.gov/compound/10071695 |
| CAP1 | https://pubchem.ncbi.nlm.nih.gov/compound/42678963 |
| CAP2 | https://pubchem.ncbi.nlm.nih.gov/compound/5193822 |
| CAP3 | https://pubchem.ncbi.nlm.nih.gov/compound/135515685 |
| Compound6 | https://pubchem.ncbi.nlm.nih.gov/compound/49786171#section=InChIKey |
| C6P1 | https://pubchem.ncbi.nlm.nih.gov/compound/70758658 |
| C6P 2 - 53090518 | https://pubchem.ncbi.nlm.nih.gov/compound/53090518 |
| C6P 3 - 53090532 | https://pubchem.ncbi.nlm.nih.gov/compound/53090532 |

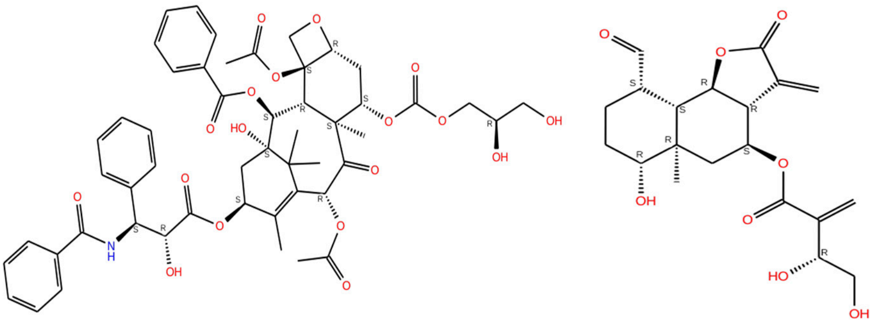

(a) (b)

*Figure S1. Chemical structures of (a) Paclitaxel and (b) Malacitanolide.*

*Table S2. ADMET profile of all the molecules used for the study.*

| **Results** | Paclitaxel | | **Results** | Malacitanolide | |
|---|---|---|---|---|---|
| **Model** | **Result** | **Probability** | **Model** | **Result** | **Probability** |
| **Absorption** | | | **Absorption** | | |
| Blood–Brain Barrier | BBB- | 0.9516 | Blood–Brain Barrier | BBB+ | 0.7086 |
| Human Intestinal Absorption | HIA+ | 0.9749 | Human Intestinal Absorption | HIA+ | 0.9295 |
| Caco-2 Permeability | Caco2- | 0.8153 | Caco-2 Permeability | Caco2- | 0.7307 |
| P-glycoprotein Substrate | Substrate | 0.8569 | P-glycoprotein Substrate | Substrate | 0.8401 |
| P-glycoprotein Inhibitor | Inhibitor<br>Non-inhibitor | 0.6178<br>0.6453 | P-glycoprotein Inhibitor | Inhibitor<br>Non-inhibitor | 0.6129<br>0.656 |
| Renal Organic Cation Transporter | Non-inhibitor | 0.9312 | Renal Organic Cation Transporter | Non-inhibitor | 0.7726 |
| **Distribution** | | | **Distribution** | | |
| Subcellular localization | Mitochondria | 0.6706 | Subcellular localization | Mitochondria | 0.8121 |
| **Metabolism** | | | **Metabolism** | | |
| CYP450 2C9 Substrate | Non-substrate | 0.8563 | CYP450 2C9 Substrate | Non-substrate | 0.8575 |

| | | | | | |
|---|---|---|---|---|---|
| CYP450 2D6 Substrate | Non-substrate | 0.8533 | CYP450 2D6 Substrate | Non-substrate | 0.8861 |
| CYP450 3A4 Substrate | Substrate | 0.6554 | CYP450 3A4 Substrate | Substrate | 0.6274 |
| CYP450 1A2 Inhibitor | Non-inhibitor | 0.8108 | CYP450 1A2 Inhibitor | Non-inhibitor | 0.8088 |
| CYP450 2C9 Inhibitor | Non-inhibitor | 0.845 | CYP450 2C9 Inhibitor | Non-inhibitor | 0.8234 |
| CYP450 2D6 Inhibitor | Non-inhibitor | 0.8949 | CYP450 2D6 Inhibitor | Non-inhibitor | 0.9388 |
| CYP450 2C19 Inhibitor | Non-inhibitor | 0.8154 | CYP450 2C19 Inhibitor | Non-inhibitor | 0.877 |
| CYP450 3A4 Inhibitor | Non-inhibitor | 0.5575 | CYP450 3A4 Inhibitor | Non-inhibitor | 0.6309 |
| CYP Inhibitory Promiscuity | Low CYP Inhibitory Promiscuity | 0.7745 | CYP Inhibitory Promiscuity | Low CYP Inhibitory Promiscuity | 0.9345 |
| **Excretion** | | | **Excretion** | | |
| **Toxicity** | | | **Toxicity** | | |
| Human Ether-a-go-go-Related Gene Inhibition | Weak inhibitor | 0.9873 | Human Ether-a-go-go-Related Gene Inhibition | Weak inhibitor | 0.9232 |
| | Non-inhibitor | 0.7263 | | Inhibitor | 0.6269 |
| AMES Toxicity | Non AMES toxic | 0.8107 | AMES Toxicity | Non AMES toxic | 0.8023 |
| Carcinogens | Non-carcinogens | 0.9121 | Carcinogens | Non-carcinogens | 0.9646 |
| Fish Toxicity | High FHMT | 0.9941 | Fish Toxicity | High FHMT | 0.9984 |
| Tetrahymena Pyriformis Toxicity | High TPT | 0.9988 | Tetrahymena Pyriformis Toxicity | High TPT | 0.997 |
| Honey Bee Toxicity | High HBT | 0.639 | Honey Bee Toxicity | High HBT | 0.7948 |
| Biodegradation | Not ready biodegradable | 0.9753 | Biodegradation | Not ready biodegradable | 0.8921 |
| Acute Oral Toxicity | III | 0.6158 | Acute Oral Toxicity | III | 0.5009 |
| Carcinogenicity (Three-class) | Non-required | 0.5139 | Carcinogenicity (Three-class) | Non-required | 0.5934 |

| Model | Value | Unit | Model | Value | Unit |
|---|---|---|---|---|---|
| **Absorption** | | | **Absorption** | | |
| Aqueous solubility | -3.6693 | LogS | Aqueous solubility | -3.8155 | LogS |
| Caco-2 Permeability | 0.1112 | LogPapp, cm/s | Caco-2 Permeability | -0.1387 | LogPapp, cm/s |
| **Distribution** | | | **Distribution** | | |
| **Metabolism** | | | **Metabolism** | | |
| **Excretion** | | | **Excretion** | | |
| **Toxicity** | | | **Toxicity** | | |
| Rat Acute Toxicity | 2.5665 | LD50, mol/kg | Rat Acute Toxicity | 2.8626 | LD50, mol/kg |
| Fish Toxicity | 0.9528 | pLC50, mg/L | Fish Toxicity | 0.5096 | pLC50, mg/L |
| Tetrahymena Pyriformis Toxicity | 0.5211 | pIGC50, ug/L | Tetrahymena Pyriformis Toxicity | 1.1509 | pIGC50, ug/L |

**Results** Canniflavin **Results** CAP1

| Model | Result | Probability | Model | Result | Probability |
|---|---|---|---|---|---|
| **Absorption** | | | **Absorption** | | |
| Blood–Brain Barrier | BBB- | 0.7896 | Blood–Brain Barrier | BBB+ | 0.9261 |
| Human Intestinal Absorption | HIA+ | 0.9857 | Human Intestinal Absorption | HIA+ | 0.9763 |
| Caco-2 Permeability | Caco2+ | 0.5279 | Caco-2 Permeability | Caco2- | 0.6624 |
| P-glycoprotein Substrate | Substrate | 0.867 | P-glycoprotein Substrate | Substrate | 0.7312 |
| P-glycoprotein Inhibitor | Inhibitor | 0.5724 | P-glycoprotein Inhibitor | Inhibitor | 0.794 |
| | Inhibitor | 0.9918 | | Non-inhibitor | 0.9105 |
| Renal Organic Cation Transporter | Non-inhibitor | 0.8499 | Renal Organic Cation Transporter | Non-inhibitor | 0.6358 |
| **Distribution** | | | **Distribution** | | |
| Subcellular localization | Mitochondria | 0.6903 | Subcellular localization | Mitochondria | 0.7731 |
| **Metabolism** | | | **Metabolism** | | |
| CYP450 2C9 Substrate | Non-substrate | 0.8127 | CYP450 2C9 Substrate | Non-substrate | 0.8052 |

| | | | | | |
|---|---|---|---|---|---|
| CYP450 2D6 Substrate | Non-substrate | 0.8376 | CYP450 2D6 Substrate | Non-substrate | 0.7611 |
| CYP450 3A4 Substrate | Substrate | 0.6995 | CYP450 3A4 Substrate | Substrate | 0.5561 |
| CYP450 1A2 Inhibitor | Inhibitor | 0.7635 | CYP450 1A2 Inhibitor | Non-inhibitor | 0.8391 |
| CYP450 2C9 Inhibitor | Inhibitor | 0.5895 | CYP450 2C9 Inhibitor | Inhibitor | 0.5 |
| CYP450 2D6 Inhibitor | Non-inhibitor | 0.6938 | CYP450 2D6 Inhibitor | Non-inhibitor | 0.7479 |
| CYP450 2C19 Inhibitor | Inhibitor | 0.6769 | CYP450 2C19 Inhibitor | Inhibitor | 0.8321 |
| CYP450 3A4 Inhibitor | Non-inhibitor | 0.5442 | CYP450 3A4 Inhibitor | Inhibitor | 0.6355 |
| CYP Inhibitory Promiscuity | High CYP Inhibitory Promiscuity | 0.7657 | CYP Inhibitory Promiscuity | High CYP Inhibitory Promiscuity | 0.5063 |
| **Excretion** | | | **Excretion** | | |
| **Toxicity** | | | **Toxicity** | | |
| Human Ether-a-go-go-Related Gene Inhibition | Weak inhibitor | 0.9033 | Human Ether-a-go-go-Related Gene Inhibition | Weak inhibitor | 0.9149 |
| | Inhibitor | 0.5 | | Inhibitor | 0.8912 |
| AMES Toxicity | Non AMES toxic | 0.8422 | AMES Toxicity | Non AMES toxic | 0.6254 |
| Carcinogens | Non-carcinogens | 0.952 | Carcinogens | Non-carcinogens | 0.8611 |
| Fish Toxicity | High FHMT | 0.9977 | Fish Toxicity | High FHMT | 0.9364 |
| Tetrahymena Pyriformis Toxicity | High TPT | 0.9992 | Tetrahymena Pyriformis Toxicity | High TPT | 0.9684 |
| Honey Bee Toxicity | High HBT | 0.7844 | Honey Bee Toxicity | Low HBT | 0.9482 |
| Biodegradation | Not ready biodegradable | 0.9437 | Biodegradation | Not ready biodegradable | 0.9967 |
| Acute Oral Toxicity | III | 0.4437 | Acute Oral Toxicity | III | 0.5329 |
| Carcinogenicity (Three-class) | Non-required | 0.7681 | Carcinogenicity (Three-class) | Non-required | 0.6466 |

| Model | Value | Unit | Model | Value | Unit |
|---|---|---|---|---|---|
| **Absorption** | | | **Absorption** | | |
| Aqueous solubility | -4.4888 | LogS | Aqueous solubility | -4.9531 | LogS |
| Caco-2 Permeability | 1.0868 | LogPapp, cm/s | Caco-2 Permeability | 1.0154 | LogPapp, cm/s |
| **Distribution** | | | **Distribution** | | |
| **Metabolism** | | | **Metabolism** | | |
| **Excretion** | | | **Excretion** | | |
| **Toxicity** | | | **Toxicity** | | |
| Rat Acute Toxicity | 3.008 | LD50, mol/kg | Rat Acute Toxicity | 2.4393 | LD50, mol/kg |
| Fish Toxicity | 0.1001 | pLC50, mg/L | Fish Toxicity | 1.555 | pLC50, mg/L |
| Tetrahymena Pyriformis Toxicity | 1.6953 | pIGC50, ug/L | Tetrahymena Pyriformis Toxicity | 0.6508 | pIGC50, ug/L |

**Results** CAP2

| Model | Result | Probability |
|---|---|---|
| **Absorption** | | |
| Blood–Brain Barrier | BBB- | 0.8101 |
| Human Intestinal Absorption | HIA+ | 0.9354 |
| Caco-2 Permeability | Caco2- | 0.7934 |
| P-glycoprotein Substrate | Substrate | 0.7131 |
| P-glycoprotein Inhibitor | Non-inhibitor | 0.8169 |
| | Non-inhibitor | 0.9187 |
| Renal Organic Cation Transporter | Non-inhibitor | 0.8992 |
| **Distribution** | | |
| Subcellular localization | Mitochondria | 0.8392 |
| **Metabolism** | | |
| CYP450 2C9 Substrate | Non-substrate | 0.7601 |

**Results** CAP3

| Model | Result | Probability |
|---|---|---|
| **Absorption** | | |
| Blood–Brain Barrier | BBB- | 0.8094 |
| Human Intestinal Absorption | HIA- | 0.5602 |
| Caco-2 Permeability | Caco2- | 0.617 |
| P-glycoprotein Substrate | Substrate | 0.5283 |
| P-glycoprotein Inhibitor | Non-inhibitor | 0.6753 |
| | Non-inhibitor | 0.7351 |
| Renal Organic Cation Transporter | Non-inhibitor | 0.8943 |
| **Distribution** | | |
| Subcellular localization | Mitochondria | 0.9075 |
| **Metabolism** | | |
| CYP450 2C9 Substrate | Non-substrate | 0.7557 |

| | | | | | |
|---|---|---|---|---|---|
| CYP450 2D6 Substrate | Non-substrate | 0.8045 | CYP450 2D6 Substrate | Non-substrate | 0.8292 |
| CYP450 3A4 Substrate | Non-substrate | 0.5827 | CYP450 3A4 Substrate | Substrate | 0.5433 |
| CYP450 1A2 Inhibitor | Non-inhibitor | 0.7065 | CYP450 1A2 Inhibitor | Non-inhibitor | 0.687 |
| CYP450 2C9 Inhibitor | Non-inhibitor | 0.8117 | CYP450 2C9 Inhibitor | Non-inhibitor | 0.7314 |
| CYP450 2D6 Inhibitor | Non-inhibitor | 0.8473 | CYP450 2D6 Inhibitor | Non-inhibitor | 0.9435 |
| CYP450 2C19 Inhibitor | Non-inhibitor | 0.6863 | CYP450 2C19 Inhibitor | Non-inhibitor | 0.6311 |
| CYP450 3A4 Inhibitor | Non-inhibitor | 0.6455 | CYP450 3A4 Inhibitor | Non-inhibitor | 0.687 |
| CYP Inhibitory Promiscuity | Low CYP Inhibitory Promiscuity | 0.7947 | CYP Inhibitory Promiscuity | Low CYP Inhibitory Promiscuity | 0.6449 |
| **Excretion Toxicity** | | | **Excretion Toxicity** | | |
| Human Ether-a-go-go-Related Gene Inhibition | Weak inhibitor | 0.9568 | Human Ether-a-go-go-Related Gene Inhibition | Weak inhibitor | 0.9888 |
| | Non-inhibitor | 0.8405 | | Non-inhibitor | 0.6848 |
| AMES Toxicity | Non AMES toxic | 0.7703 | AMES Toxicity | AMES toxic | 0.6863 |
| Carcinogens | Non-carcinogens | 0.8729 | Carcinogens | Non-carcinogens | 0.6349 |
| Fish Toxicity | High FHMT | 0.9896 | Fish Toxicity | High FHMT | 0.9913 |
| Tetrahymena Pyriformis Toxicity | High TPT | 0.9897 | Tetrahymena Pyriformis Toxicity | High TPT | 0.9903 |
| Honey Bee Toxicity | Low HBT | 0.635 | Honey Bee Toxicity | Low HBT | 0.8474 |
| Biodegradation | Not ready biodegradable | 0.8843 | Biodegradation | Not ready biodegradable | 1 |
| Acute Oral Toxicity | III | 0.693 | Acute Oral Toxicity | III | 0.7267 |
| Carcinogenicity (Three-class) | Non-required | 0.6488 | Carcinogenicity (Three-class) | Non-required | 0.5953 |

| Model | Value | Unit | Model | Value | Unit |
|---|---|---|---|---|---|
| **Absorption** | | | **Absorption** | | |
| Aqueous solubility | -3.732 | LogS | Aqueous solubility | -3.2752 | LogS |
| Caco-2 Permeability | 0.0204 | LogPapp, cm/s | Caco-2 Permeability | 0.244 | LogPapp, cm/s |
| **Distribution Metabolism Excretion Toxicity** | | | **Distribution Metabolism Excretion Toxicity** | | |
| Rat Acute Toxicity | 2.2197 | LD50, mol/kg | Rat Acute Toxicity | 2.1684 | LD50, mol/kg |
| Fish Toxicity | 1.3924 | pLC50, mg/L | Fish Toxicity | 0.8673 | pLC50, mg/L |
| Tetrahymena Pyriformis Toxicity | 0.1371 | pIGC50, ug/L | Tetrahymena Pyriformis Toxicity | 0.4742 | pIGC50, ug/L |

**Results** Compound 6 **Results** C6P1

| Model | Result | Probability | Model | Result | Probability |
|---|---|---|---|---|---|
| **Absorption** | | | **Absorption** | | |
| Blood–Brain Barrier | BBB+ | 0.9621 | Blood–Brain Barrier | BBB+ | 0.989 |
| Human Intestinal Absorption | HIA+ | 1 | Human Intestinal Absorption | HIA+ | 1 |
| Caco-2 Permeability | Caco2+ | 0.5 | Caco-2 Permeability | Caco2- | 0.6189 |
| P-glycoprotein Substrate | Non-substrate | 0.7387 | P-glycoprotein Substrate | Non-substrate | 0.7411 |
| P-glycoprotein Inhibitor | Non-inhibitor | 0.7691 | P-glycoprotein Inhibitor | Non-inhibitor | 0.6909 |
| | Inhibitor | 0.8238 | | Inhibitor | 0.8721 |
| Renal Organic Cation Transporter | Non-inhibitor | 0.5792 | Renal Organic Cation Transporter | Non-inhibitor | 0.8151 |
| **Distribution** | | | **Distribution** | | |
| Subcellular localization | Mitochondria | 0.5089 | Subcellular localization | Mitochondria | 0.4231 |
| **Metabolism** | | | **Metabolism** | | |
| CYP450 2C9 Substrate | Non-substrate | 0.8048 | CYP450 2C9 Substrate | Non-substrate | 0.8451 |

| | | | | | |
|---|---|---|---|---|---|
| CYP450 2D6 Substrate | Non-substrate | 0.8363 | CYP450 2D6 Substrate | Non-substrate | 0.8295 |
| CYP450 3A4 Substrate | Non-substrate | 0.6189 | CYP450 3A4 Substrate | Non-substrate | 0.5722 |
| CYP450 1A2 Inhibitor | Inhibitor | 0.9579 | CYP450 1A2 Inhibitor | Inhibitor | 0.9167 |
| CYP450 2C9 Inhibitor | Inhibitor | 0.5763 | CYP450 2C9 Inhibitor | Non-inhibitor | 0.5159 |
| CYP450 2D6 Inhibitor | Inhibitor | 0.7205 | CYP450 2D6 Inhibitor | Non-inhibitor | 0.5942 |
| CYP450 2C19 Inhibitor | Inhibitor | 0.9172 | CYP450 2C19 Inhibitor | Inhibitor | 0.7478 |
| CYP450 3A4 Inhibitor | Non-inhibitor | 0.5634 | CYP450 3A4 Inhibitor | Inhibitor | 0.7197 |
| CYP Inhibitory Promiscuity | High CYP Inhibitory Promiscuity | 0.9361 | CYP Inhibitory Promiscuity | High CYP Inhibitory Promiscuity | 0.9042 |
| **Excretion** | | | **Excretion** | | |
| **Toxicity** | | | **Toxicity** | | |
| Human Ether-a-go-go-Related Gene Inhibition | Weak inhibitor | 0.8986 | Human Ether-a-go-go-Related Gene Inhibition | Weak inhibitor | 0.9387 |
| | Non-inhibitor | 0.7098 | | Non-inhibitor | 0.658 |
| AMES Toxicity | Non AMES toxic | 0.6462 | AMES Toxicity | Non AMES toxic | 0.6507 |
| Carcinogens | Non-carcinogens | 0.8941 | Carcinogens | Non-carcinogens | 0.7336 |
| Fish Toxicity | High FHMT | 0.9769 | Fish Toxicity | Low FHMT | 0.7164 |
| Tetrahymena Pyriformis Toxicity | High TPT | 0.9957 | Tetrahymena Pyriformis Toxicity | High TPT | 0.6978 |
| Honey Bee Toxicity | Low HBT | 0.7296 | Honey Bee Toxicity | Low HBT | 0.8259 |
| Biodegradation | Not ready biodegradable | 1 | Biodegradation | Not ready biodegradable | 0.9943 |
| Acute Oral Toxicity | III | 0.6582 | Acute Oral Toxicity | III | 0.4803 |
| Carcinogenicity (Three-class) | Non-required | 0.5758 | Carcinogenicity (Three-class) | Non-required | 0.4774 |

| Model | Value | Unit | Model | Value | Unit |
|---|---|---|---|---|---|
| **Absorption** | | | **Absorption** | | |
| Aqueous solubility | -4.2569 | LogS | Aqueous solubility | -2.8923 | LogS |
| Caco-2 Permeability | 1.2981 | LogPapp, cm/s | Caco-2 Permeability | 0.6493 | LogPapp, cm/s |
| **Distribution Metabolism Excretion Toxicity** | | | **Distribution Metabolism Excretion Toxicity** | | |
| Rat Acute Toxicity | 2.5492 | LD50, mol/kg | Rat Acute Toxicity | 2.6862 | LD50, mol/kg |
| Fish Toxicity | 1.0463 | pLC50, mg/L | Fish Toxicity | 1.7972 | pLC50, mg/L |
| Tetrahymena Pyriformis Toxicity | 1.0204 | pIGC50, ug/L | Tetrahymena Pyriformis Toxicity | 0.3591 | pIGC50, ug/L |

## Results C6P2 / Results C6P3

| Model | Result | Probability | Model | Result | Probability |
|---|---|---|---|---|---|
| **Absorption** | | | **Absorption** | | |
| Blood–Brain Barrier | BBB+ | 0.9862 | Blood–Brain Barrier | BBB+ | 0.9934 |
| Human Intestinal Absorption | HIA+ | 1 | Human Intestinal Absorption | HIA+ | 1 |
| Caco-2 Permeability | Caco2- | 0.5441 | Caco-2 Permeability | Caco2+ | 0.5 |
| P-glycoprotein Substrate | Non-substrate | 0.5996 | P-glycoprotein Substrate | Non-substrate | 0.6894 |
| P-glycoprotein Inhibitor | Non-inhibitor | 0.6082 | P-glycoprotein Inhibitor | Non-inhibitor | 0.6123 |
| | Inhibitor | 0.6555 | | Inhibitor | 0.5491 |
| Renal Organic Cation Transporter | Non-inhibitor | 0.801 | Renal Organic Cation Transporter | Non-inhibitor | 0.7979 |
| **Distribution** | | | **Distribution** | | |
| Subcellular localization | Mitochondria | 0.4963 | Subcellular localization | Mitochondria | 0.479 |
| **Metabolism** | | | **Metabolism** | | |
| CYP450 2C9 Substrate | Non-substrate | 0.871 | CYP450 2C9 Substrate | Non-substrate | 0.845 |
| CYP450 2D6 Substrate | Non-substrate | 0.7958 | CYP450 2D6 Substrate | Non-substrate | 0.8253 |

| | | | | | |
|---|---|---|---|---|---|
| CYP450 3A4 Substrate | Substrate | 0.6641 | CYP450 3A4 Substrate | Substrate | 0.5426 |
| CYP450 1A2 Inhibitor | Inhibitor | 0.7879 | CYP450 1A2 Inhibitor | Inhibitor | 0.8754 |
| CYP450 2C9 Inhibitor | Inhibitor | 0.6187 | CYP450 2C9 Inhibitor | Non-inhibitor | 0.5609 |
| CYP450 2D6 Inhibitor | Non-inhibitor | 0.6673 | CYP450 2D6 Inhibitor | Non-inhibitor | 0.7679 |
| CYP450 2C19 Inhibitor | Inhibitor | 0.8867 | CYP450 2C19 Inhibitor | Inhibitor | 0.751 |
| CYP450 3A4 Inhibitor | Inhibitor | 0.8744 | CYP450 3A4 Inhibitor | Inhibitor | 0.7109 |
| CYP Inhibitory Promiscuity | High CYP Inhibitory Promiscuity | 0.8892 | CYP Inhibitory Promiscuity | High CYP Inhibitory Promiscuity | 0.8657 |
| **Excretion Toxicity** | | | **Excretion Toxicity** | | |
| Human Ether-a-go-go-Related Gene Inhibition | Weak inhibitor | 0.8989 | Human Ether-a-go-go-Related Gene Inhibition | Weak inhibitor | 0.8525 |
| | Non-inhibitor | 0.7399 | | Non-inhibitor | 0.8205 |
| AMES Toxicity | AMES toxic | 0.5335 | AMES Toxicity | Non AMES toxic | 0.5357 |
| Carcinogens | Non-carcinogens | 0.7909 | Carcinogens | Non-carcinogens | 0.6942 |
| Fish Toxicity | Low FHMT | 0.7426 | Fish Toxicity | Low FHMT | 0.913 |
| Tetrahymena Pyriformis Toxicity | High TPT | 0.7975 | Tetrahymena Pyriformis Toxicity | High TPT | 0.844 |
| Honey Bee Toxicity | Low HBT | 0.7096 | Honey Bee Toxicity | Low HBT | 0.8417 |
| Biodegradation | Not ready biodegradable | 1 | Biodegradation | Not ready biodegradable | 0.9904 |
| Acute Oral Toxicity | III | 0.6329 | Acute Oral Toxicity | III | 0.6467 |
| Carcinogenicity (Three-class) | Non-required | 0.5322 | Carcinogenicity (Three-class) | Non-required | 0.4972 |
| **Model** | **Value** | **Unit** | **Model** | **Value** | **Unit** |
| **Absorption** | | | **Absorption** | | |
| Aqueous solubility | -3.2005 | LogS | Aqueous solubility | -3.1336 | LogS |
| Caco-2 Permeability | 1.035 | LogPapp, cm/s | Caco-2 Permeability | 1.2294 | LogPapp, cm/s |
| **Distribution Metabolism Excretion Toxicity** | | | **Distribution Metabolism Excretion Toxicity** | | |
| Rat Acute Toxicity | 2.537 | LD50, mol/kg | Rat Acute Toxicity | 2.5253 | LD50, mol/kg |
| Fish Toxicity | 1.2542 | pLC50, mg/L | Fish Toxicity | 1.673 | pLC50, mg/L |
| Tetrahymena Pyriformis Toxicity | 0.3375 | pIGC50, ug/L | Tetrahymena Pyriformis Toxicity | 0.4582 | pIGC50, ug/L |

## Other information:

The 100ns Molecular Dynamics simulations consisted of 5002 frames per trajectory. Simulation Interaction Diagram Reports produced by the Schrodinger Maestro software consisted of Protein Information, Ligand Information, Protein-Ligand RMSD, Protein RMSF, Protein Secondary Structure information, Ligand RMSF, Protein–Ligand Contacts, Ligand–Protein Contacts, Ligand Torsion Profile and other ligand properties. These reports for all the molecules have been uploaded as supplementary information. Further, the MM-GBSA values calculated for each trajectory have been uploaded as individual .csv files. The 100ns molecular dynamics trajectory videos were captured for the ligand–receptor complex and are provided as supplementary information. Further, the Ligand RMSD raw data file is recorded in the file titled Lig_wrt_Prot_RMSD.xlsx

Additionally, predicted toxicity reports obtained from the ProTox 3.0 server for all molecules are included. All of this additional information has been uploaded to the Zenodo repository, accessible using the following link: https://zenodo.org/records/14004458

**BLI Results**

*Table S3. BLI results summary.*

| Receptor | Ligand | Assay std (Y/N) | Dose Dependence | Data points excluded | Flipped? | KD | Ka | Kdis |
|---|---|---|---|---|---|---|---|---|
| DENV1 | Paclitaxcel_trial1 | Y | Y | N | N | 5.14E-10 | 9.51E+02 | 4.88E-07 |
| DENV1 | Paclitaxcel_trial2 | Y | Y | N | N | 7.68E-10 | 6.36E+02 | 4.88E-07 |
| DENV1 | CAP1_trial1 | Y | Y | N | N | 8.07E-07 | 2.50E+03 | 2.02E-03 |
| DENV1 | CAP1_trial2 | Y | Y | N | N | 6.67E-07 | 5.01E+03 | 3.34E-03 |
| DENV2 | Paclitaxcel_trial1 | Y | N | 12.5 and 6.25 | N | 1.07E-09 | 4.58E+02 | 4.88E-07 |
| DENV2 | Paclitaxcel_trial2 | Y | Y | N | N | 2.62E-10 | 1.87E+03 | 4.88E-07 |
| DENV2 | CAP1_trial1 | Y | N | 25 | N | 4.13E-04 | 6.60E+02 | 2.73E-01 |
| DENV2 | CAP1_trial2 | Y | N | 25 and 12.5 | ALL | 2.33E-03 | 5.15E+01 | 1.20E-01 |

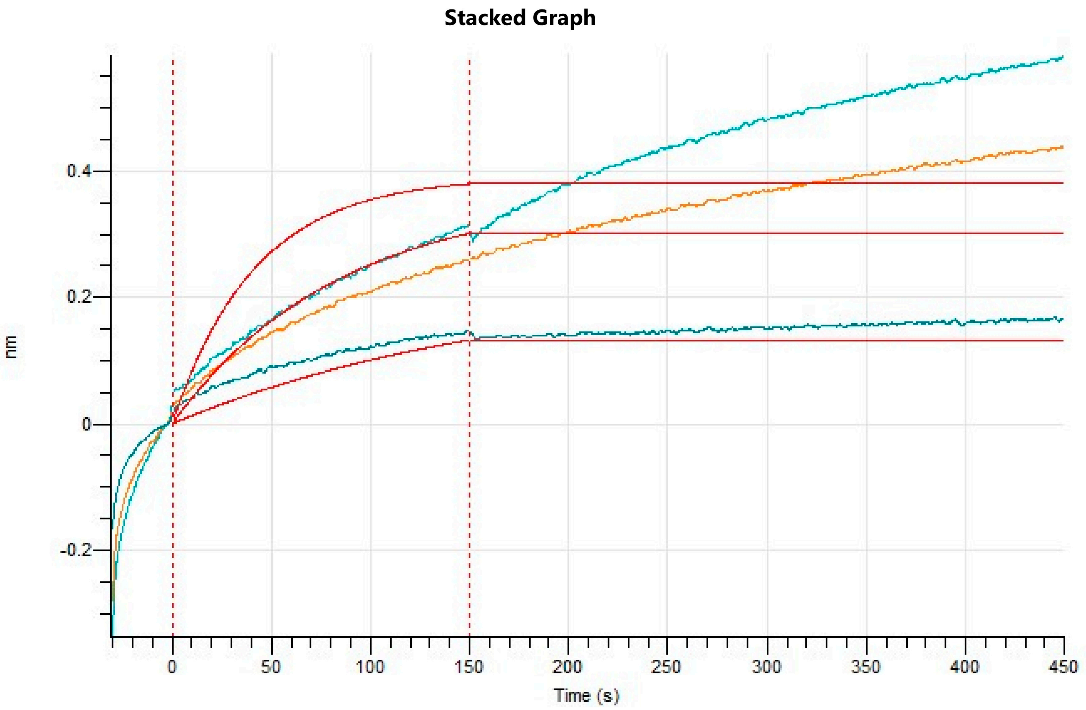


| Index | Color | Assoc. (Sample) Loc. | Sample ID | Dissoc. Loc. | Conc. (uM) | Response | KD (M) | ka (1/Ms) | kdis (1/s) | Rmax | kobs (1/s) | Req |
|---|---|---|---|---|---|---|---|---|---|---|---|---|
| 0 | | p1F4 | | p1F3 | 25 | 0.3048 | 5.136E-10 | 9.508E02 | 4.883E-07 | 0.391 | 2.377E-02 | 0.391 |
| 1 | | p1G4 | | p1G3 | 12.5 | 0.2521 | 5.136E-10 | 9.508E02 | 4.883E-07 | 0.3625 | 1.189E-02 | 0.3625 |
| 2 | | p1H4 | | p1H3 | 6.25 | 0.1416 | 5.136E-10 | 9.508E02 | 4.883E-07 | 0.2247 | 5.943E-03 | 0.2247 |

*Figure S2. DENV1- Paclitaxel trial 1 BLI assay.*

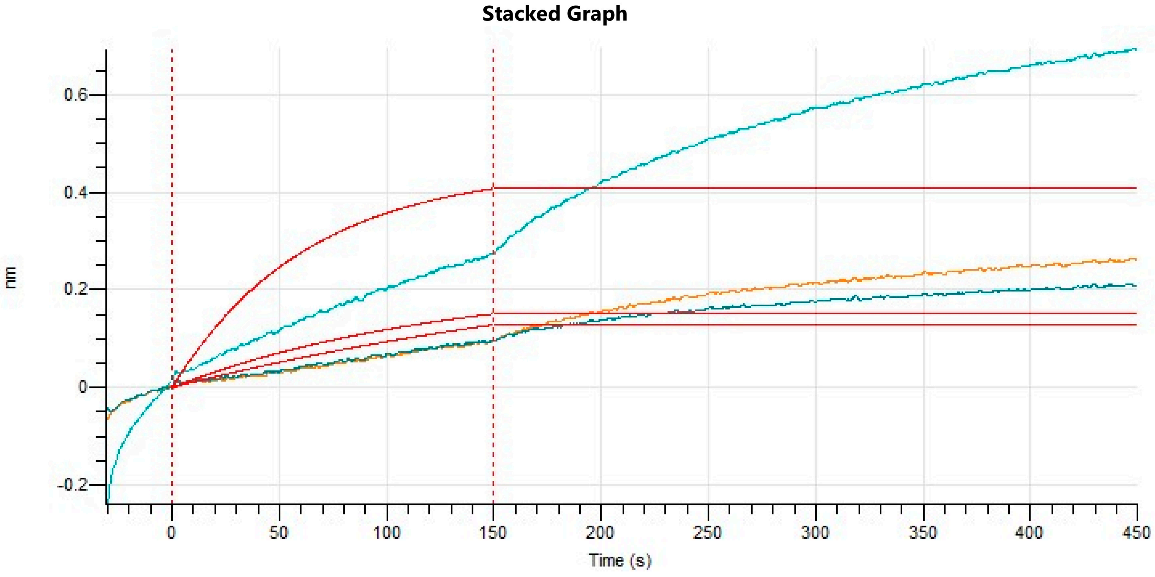


| Index | Color | Assoc. (Sample) Loc. | Sample ID | Dissoc. Loc. | Conc. (uM) | Response | KD (M) | ka (1/Ms) | kdis (1/s) | Rmax | kobs (1/s) | Req |
|---|---|---|---|---|---|---|---|---|---|---|---|---|
| 0 | | p1B4 | | p1B3 | 25 | 0.2633 | 7.680E-10 | 6.357E02 | 4.883E-07 | 0.4489 | 1.589E-02 | 0.4489 |
| 1 | | p1C4 | | p1C3 | 12.5 | 0.0915 | 7.680E-10 | 6.357E02 | 4.883E-07 | 0.2171 | 7.947E-03 | 0.2171 |
| 2 | | p1D4 | | p1D3 | 6.25 | 0.0934 | 7.680E-10 | 6.357E02 | 4.883E-07 | 0.2871 | 3.974E-03 | 0.2871 |

*Figure S3. DENV1- Paclitaxel trial 2 BLI assay.*

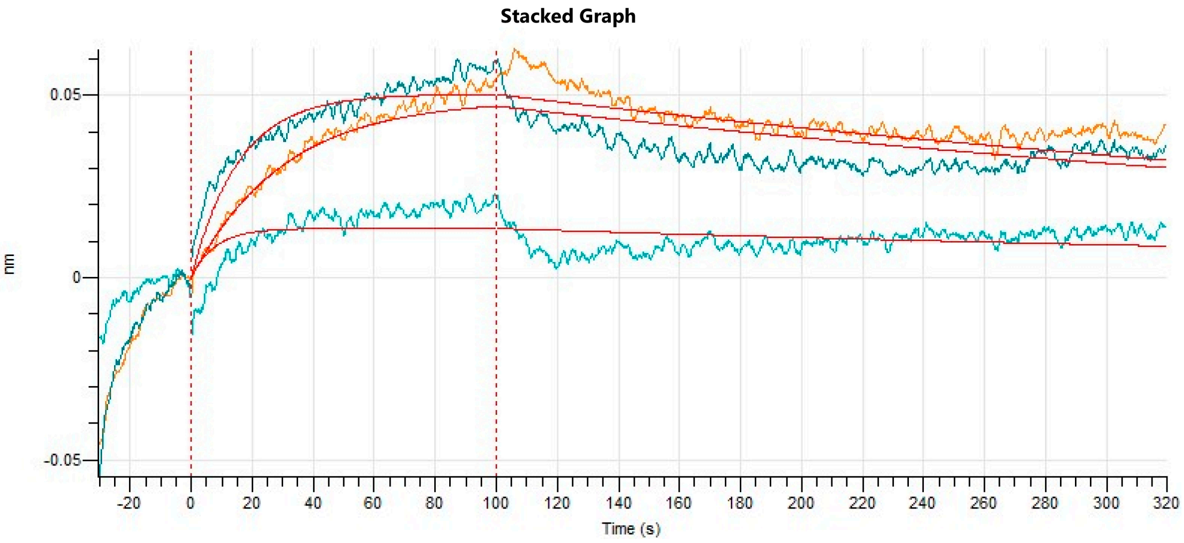


| Index | Color | Assoc. (Sample) Loc. | Sample ID | Dissoc. Loc. | Conc. (uM) | Response | KD (M) | ka (1/Ms) | kdis (1/s) | Rmax | kobs (1/s) | Req |
|---|---|---|---|---|---|---|---|---|---|---|---|---|
| 0 | | p1B10 | | p1B9 | 50 | 0.0202 | 4.153E-07 | 6.148E03 | 2.553E-03 | 0.0136 | 3.100E-01 | 0.0135 |
| 1 | | p1C10 | | p1C9 | 25 | 0.0524 | | | | | | |
| 2 | | p1D10 | | p1D9 | 12.5 | 0.0578 | 4.153E-07 | 6.148E03 | 2.553E-03 | 0.0484 | 7.941E-02 | 0.0469 |

*Figure S4. DENV1- CAP1 trial 1 BLI assay.*

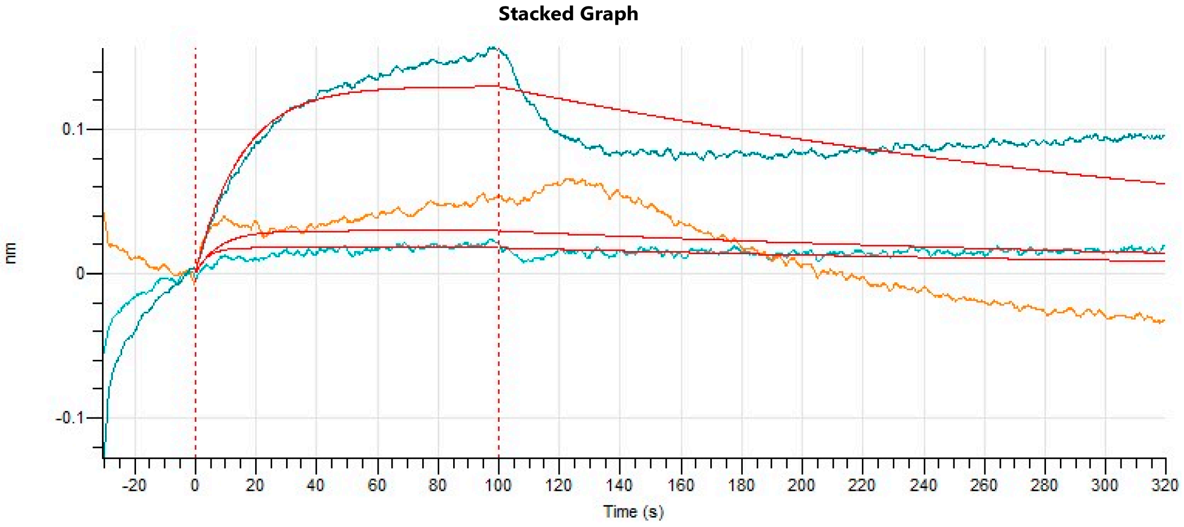


| Index | Color | Assoc. (Sample) Loc. | Sample ID | Dissoc. Loc. | Conc. (uM) | Response | KD (M) | ka (1/Ms) | kdis (1/s) | Rmax | kobs (1/s) | Req |
|---|---|---|---|---|---|---|---|---|---|---|---|---|
| 0 | | p1F4 | | p1F3 | 50 | 0.0196 | 6.674E-07 | 5.008E03 | 3.343E-03 | 0.0181 | 2.538E-01 | 0.0179 |
| 1 | | p1G4 | | p1G3 | 25 | 0.0507 | 6.674E-07 | 5.008E03 | 3.343E-03 | 0.0304 | 1.285E-01 | 0.0296 |
| 2 | | p1H4 | | p1H3 | 12.5 | 0.1503 | 6.674E-07 | 5.008E03 | 3.343E-03 | 0.1368 | 6.595E-02 | 0.1299 |

*Figure S5. DENV1- CAP1 trial 2 BLI assay.*

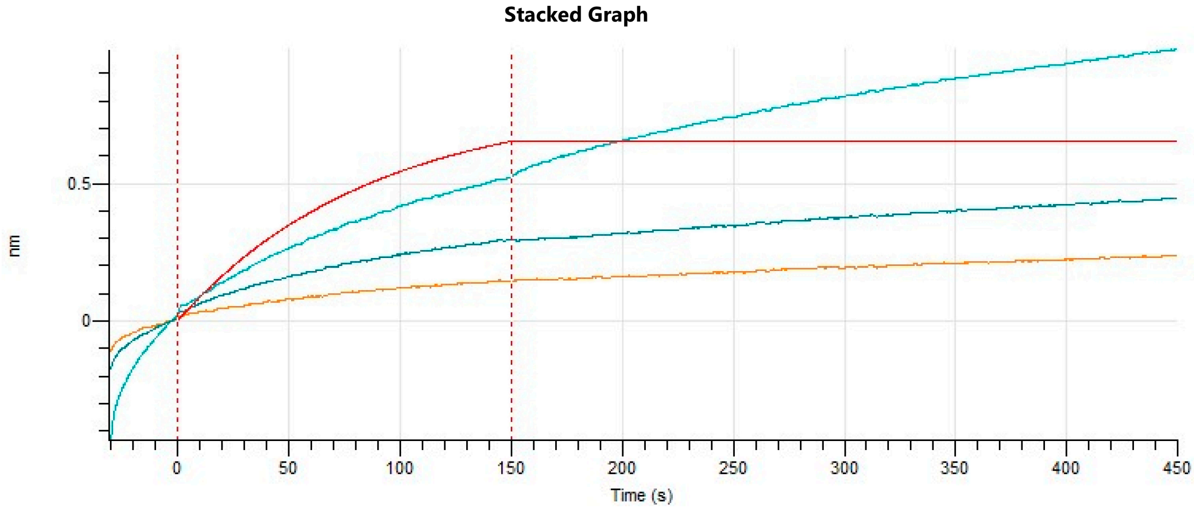


| Index | Color | Assoc. (Sample) Loc. | Sample ID | Dissoc. Loc. | Conc. (uM) | Response | KD (M) | ka (1/Ms) | kdis (1/s) | Rmax | kobs (1/s) | Req |
|---|---|---|---|---|---|---|---|---|---|---|---|---|
| 0 | | p1B4 | | p1B3 | 25 | 0.5096 | 8.687E-10 | 4.213E02 | 3.660E-07 | 0.8872 | 1.053E-02 | 0.8871 |
| 1 | | p1C4 | | p1C3 | 12.5 | 0.1452 | 8.687E-10 | 4.213E02 | 3.660E-07 | 0.3234 | 5.267E-03 | 0.3233 |
| 2 | | p1D4 | | p1D3 | 6.25 | 0.2908 | 8.687E-10 | 4.213E02 | 3.660E-07 | 1.0551 | 2.633E-03 | 1.055 |

*Figure S6. DENV2- Paclitaxel trial 1 BLI assay.*

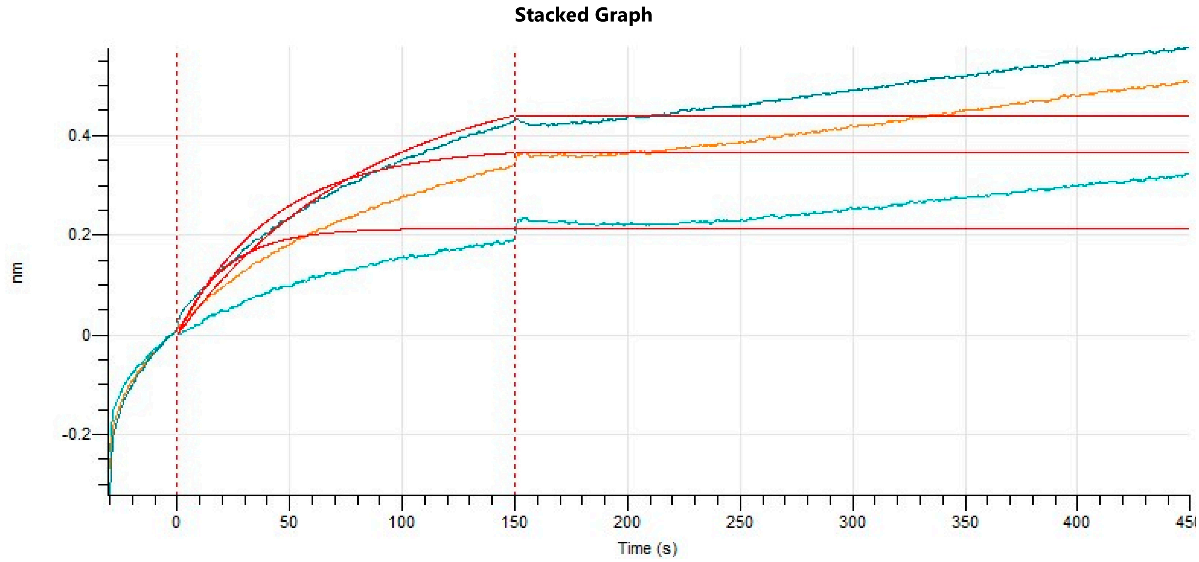


| Index | Color | Assoc. (Sample) Loc. | Sample ID | Dissoc. Loc. | Conc. (uM) | Response | KD (M) | ka (1/Ms) | kdis (1/s) | Rmax | kobs (1/s) | Req |
|---|---|---|---|---|---|---|---|---|---|---|---|---|
| 0 | | p1B10 | | p1B9 | 25 | 0.3641 | 1.623E-10 | 3.008E03 | 4.883E-07 | 0.3066 | 7.521E-02 | 0.3066 |
| 1 | | p1C10 | | p1C9 | 12.5 | 0.3069 | 1.623E-10 | 3.008E03 | 4.883E-07 | 0.272 | 3.760E-02 | 0.272 |
| 2 | | p1D10 | | p1D9 | 6.25 | 0.4326 | 1.623E-10 | 3.008E03 | 4.883E-07 | 0.4631 | 1.880E-02 | 0.463 |

*Figure S7. DENV2- Paclitaxel trial 2 BLI assay.*

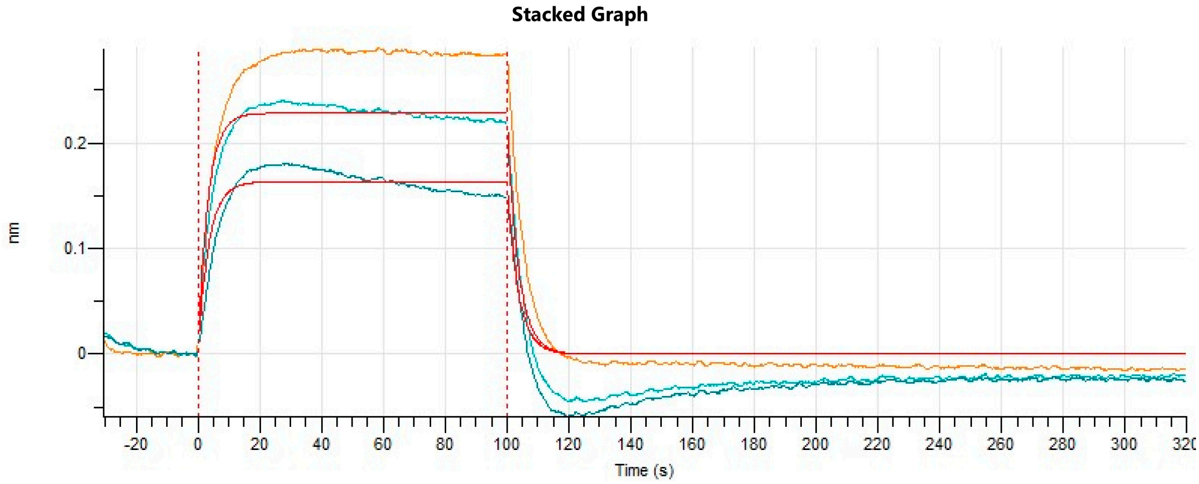


| Index | Color | Assoc. (Sample) Loc. | Sample ID | Dissoc. Loc. | Conc. (uM) | Response | KD (M) | ka (1/Ms) | kdis (1/s) | Rmax | kobs (1/s) | Req |
|---|---|---|---|---|---|---|---|---|---|---|---|---|
| 0 | | p1B10 | | p1B9 | 50 | 0.2213 | 1.472E-03 | 1.624E02 | 2.390E-01 | 6.9777 | 2.471E-01 | 0.2293 |
| 1 | | p1C10 | | p1C9 | 25 | 0.283 | 1.472E-03 | 1.624E02 | 2.390E-01 | 17.0003 | 2.430E-01 | 0.284 |
| 2 | | p1D10 | | p1D9 | 12.5 | 0.15 | 1.472E-03 | 1.624E02 | 2.390E-01 | 19.437 | 2.410E-01 | 0.1637 |

*Figure S8. DENV2- CAP1 trial 1 BLI assay.*

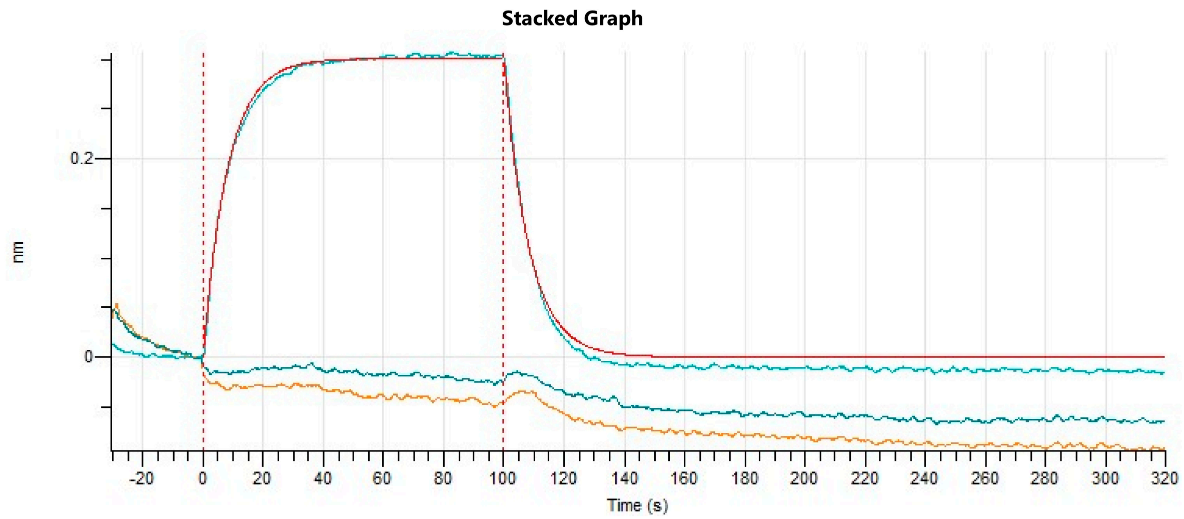


| Index | Color | Assoc. (Sample) Loc. | Sample ID | Dissoc. Loc. | Conc. (uM) | Response | KD (M) | ka (1/Ms) | kdis (1/s) | Rmax | kobs (1/s) | Req |
|---|---|---|---|---|---|---|---|---|---|---|---|---|
| 0 | | p1F10 | | p1F9 | 50 | 0.3036 | 4.789E-04 | 2.432E02 | 1.165E-01 | 3.1713 | 1.286E-01 | 0.2998 |
| 1 | | p1G10 | | p1G9 | 25 | -4.505E-02 | 4.789E-04 | 2.432E02 | 1.165E-01 | 0 | 1.226E-01 | 0 |
| 2 | | p1H10 | | p1H9 | 12.5 | -2.483E-02 | 4.789E-04 | 2.432E02 | 1.165E-01 | 0 | 1.195E-01 | 0 |

*Figure S9. DENV2- CAP1 trial 2 BLI assay.*
